# Digital Health Behavioural Interventions to Support Physical Activity and Sedentary Behaviour in Adults after Stroke: A Systematic Literature Review with Meta-Analysis of Controlled Trials

**DOI:** 10.3390/bs13010062

**Published:** 2023-01-10

**Authors:** Serena Caitlin Yen Wang, Aikaterini Kassavou

**Affiliations:** 1 Harvard Medical School, Boston, MA 02115, USA; 2 Department of Public Health and Primary Care, University of Cambridge, Cambridge CB2 0SR, UK

**Keywords:** digital intervention, stroke, prevention, treatment, systematic review, meta-analysis

## Abstract

*Background:* As the global prevalence of stroke continues to rise, it becomes increasingly pressing to investigate digital health behaviour change interventions that promote physical activity and reduce sedentary behaviour for stroke patients to support active lifestyles. *Purpose:* The primary aim of this study is to investigate the effectiveness of digital health interventions in promoting physical activity and reducing sedentary behaviour for stroke patients. The secondary aim is to investigate the intervention components that explain intervention effectiveness to further inform intervention development and policy making. *Methods:* A systematic search of the literature was conducted in four databases (Scopus, MEDLINE (PubMed), Web of Science, and PsychINFO) to identify the most robust evidence in the form of randomised controlled trials of digital interventions for patients with stroke. A random-effects meta-analysis were utilized to quantify the intervention effects on behaviour change, and subgroup analyses to characterise intervention effective components. *Results:* In total, 16 RCTs were deemed eligible and included in the systematic review. Meta-analyses suggested significant improvements in physical activity (SMD = 0.39, 95% CI 0.17, 0.61, N = 326, *p* < 0.001, I_2_ = 0%), and reductions in time of sedentary behaviour (SMD= −0.45, 95% CI −0.76, -0.14, N = 167, *p* = 0.00, I_2_ = 0%) after stroke. The 10 m walk test for physical activity, and the timed up and go test for sedentary behaviour, were the objective outcome measures in the most effective behavioural change interventions. Subgroup analyses found that most effective interventions were underpinned by theories of self-regulation and utilised interactive functions to engage patients with the processes of behaviour change. *Conclusions:* Digital self-monitoring behavioural interventions are effective in promoting physical activity for stroke patients in adjunct to usual care clinical practice and rehabilitation programmes. Rigorous studies are required to provide evidence to disentangle the most effective intervention components for preventative practices and rehabilitation programs and to inform policymaking for stroke treatment.

## 1. Introduction

With an increasingly ageing population worldwide, stroke has become a leading cause of death and a major cause of disability, annually impacting 15 million people globally [1,2]. In the UK, stroke is the third most common cause of death and a main cause of acquired disability [3]. In the United States, the ageing of the population is expected to increase the prevalence of stroke by 3.4 million people between 2012 and 2030 [4,5]. The morbidity associated with stroke has been estimated to cost of £28 billion per year for medication, healthcare services, and missed days of work [4,6].

Improved physical activity by gradually reducing sedentary behaviour after stroke, has a well-established evidence base for their benefits in minimizing cardio-cerebral risk. Numerous studies have found an inverse relationship between physical activity and stroke risk [7,8,9,10,11,12,13]. Importantly, recent reviews estimate that physical activity is associated with a 25–30% risk reduction for stroke [14,15].

Physical activity is a form of exercise and whole-body movement that is necessary for patients with stroke, and can be done at any level of skills [16]. The rehabilitative qualities of physical activity improve functionality since fitness is greatly reduced in people after stroke when compared to age-matched counterparts [17]. The discrepancy in fitness occurs because many stroke survivors are left with residual impairments such as poor balance and decreased muscle strength that makes physical activity more challenging and the risks of falls whilst exercising more frequent [18].

Sedentary behaviour is therefore most prevalent in people after stroke, and it refers to any walking behaviour that has an energy expenditure less than or equal to 1.5 metabolic equivalents (METs). Sedentary behaviour includes activities conducted when sitting or lying down such as viewing television, using the computer, playing games, and driving automobiles. Stroke survivors tend to be more sedentary compared to their healthy counterparts for similar reasons why they tend to exercise less [19]. Thus, reducing sedentary time is important as it typically involves replacing it with some form of physical activity body movement—even including the demands of simply rising from sitting on a chair. Sit-to-stand transitions increase metabolic energy expenditure by 35% above resting levels [20] and utilize 78% to 97% of maximal muscle strength in older adults [21]. Therefore, the most essential element of interventions to reduce and fragment sitting time in patients after stroke could result in benefits that resemble those from physical activity and even exercise.

Despite the striking benefits of this behaviour, the implementation of physical activity treatment programs for stroke patients is complex, and it requires many resources and face-to-face sessions. Currently, there is a lack of evidence about effective and cost-effective interventions to support patients after stroke to improve physical activity and sedentary behaviour. Thus, behavioural interventions that improve physical activity after stroke by interrupting and fragmenting the prolonged periods of sedentary behaviours need to be explicitly characterized to better inform usual care rehabilitation programs.

Digital health interventions have the potential to bridge these treatment and intervention gaps. With the new wave of digital health technologies that have emerged, support for behaviour change is more accessible and has a wider reach, while further engaging users in an effective, low-cost, and personalized format with their self-care. Access to these technologies is increasing around the globe with internet usage as high as 95% in most developed countries and 60% worldwide [22]. Especially with the COVID-19 pandemic pushing medical treatment onto digital platforms, telemedicine and telehealth have become increasingly popular as a way to receive high-quality and expert advice for healthcare. Thus, digital health technologies have a significant advantage to facilitate health behaviour change interventions to support stroke patients increase physical activity and reduce sedentary behaviour.

There are various types of digital technologies that proved feasible to facilitate health behaviour change interventions, such as, but not limited to, SMS text messaging, mobile phone applications, online computer websites, motor sensor devices, wearables, or virtual reality devices. These digital interventions require participants’ use and interaction with the digital modalities; they are two-way interactive intervention, or one-way interventions that are utilised for information provision only. They also might consist of several behaviour change strategies that aim to modify the underlying mechanism of behaviour change and thus bring about the intended effects of interest. For example, there is an evidence base suggesting that behaviour change interventions targeted at the individual are more likely to produce sustained changes on health behaviours when they support individuals to self-regulate their health behaviours [23,24,25]. Self-monitoring interventions usually entail recording or self-recording behavioural performance, providing feedback on behaviour or enabling self-observation in real time or retrospectively, and adjusting setting goals based on performance [23]. These strategies are hypothesised to motivate and enable individuals to self-regulate and exercise control over their health behaviour change; and they have shown promise to support both physical activity and sedentary behaviour in adult populations with long-term health conditions [24].

However, there is a gap in the literature of a rigorous evidence base that has examined digital health interventions that support physical activity and sedentary behaviour for stroke patients. Previous reviews have looked at components of this evidence sporadically. For example, previous studies investigated the associations between digital health interventions and physical activity or sedentary behaviour—finding a positive relationship in improving each or both of these behaviours. However, these interventions were neither specific for stroke patients nor precise regarding their preventative or treatment mechanisms and effects within healthcare [26,27,28,29]. There are also systematic reviews that focus on physical activity interventions for stroke patients without focusing on the digital health aspects that are especially prevalent in our increasingly technologically advanced society [30,31]. One review found that it is possible to modify daily physical activity levels and sedentary behaviour poststroke but there is insufficient evidence to suggest effectiveness of a particular intervention or combination of interventions among this group of patients [30]. To our knowledge, this systematic review of randomized controlled trials is the first and most up-to-date evidence that investigates remote, digitally automated, behavioural interventions to support and improve physical activity and reduce sedentary behaviour for stroke patients. This review will fill the gap in the literature and provides a pertinent understanding of the evolving digital landscape on health behaviour change interventions to support physical activity for stroke patients. Specifically, this review will quantify the effectiveness of digital health interventions to support physical activity and sedentary behaviour, and it will identify the effective intervention components that support improvements in physical activity for stroke patients.

## 2. Materials and Methods

The review protocol was registered on PROSPERO (Registration Number: CRD42022330303) by S.W. [32]. The review further adheres to the Preferred Reporting Items for Systematic reviews and Meta-Analyses PRISMA guidelines and checklist [33].

### 2.1. Study Eligibility

The PICOS framework was used to define the study question and identify eligible studies. This systematic review focused on Randomised Controlled Trials (RCTs) only, and studies were included if they fulfilled the following criteria (see also in Appendix A Table A1).

The population of interest is adults, who were at least 18 years old and had experienced a stroke. There should be an explicit report about interventions being designed for adults with a diagnosis of stroke, for the eligible population of this review. Although there were no limitations as to when and how the stroke struck for patients, interventions that focused on only early-stage post-stroke and rehabilitation were excluded since the focus of this review was to evaluate digital interventions for long-term treatment.

Eligible behavioural interventions for inclusion were required to target changes in physical inactivity and sedentary behaviour, consisting of strategies to promote self-regulation, and facilitated via digital automated mediums such as, but not limited to, text messaging, mobile phone applications, or online computer sites. Examples include smartphone apps and SMS text message services that provide feedback on the amount of physical activity performed, combined with advice, motivation, information, or support to improve physical activity. Other interventions could include online and offline digital games or interactive apps accessed by a computer or handheld device that encourage physical activity or provide mobility feedback to motivate and change sedentary behaviour. Technologies that are adapted for individual-led mobile at-home, personal usage such as virtual reality devices, motor sensor devices, and wearable sensors were included.

Technology-controlled or technology-assisted interventions were excluded. That is, interventions that utilized technology or robotic mechanisms to assist control of bodily movement at early stages of stroke rehabilitation or for re-gaining functionality and mobility after stroke were excluded.

Randomised controlled trials (RCT) only were included in this review to emphasize rigorous methodological quality for developing this evidence-base. The RCT design considers both measured and unmeasured confounding variables and minimizes potential biases compared to observational studies [34]. Studies must utilize an adequate randomisation process and method to be considered an RCT; thus, those that used inadequate randomisation processes are considered quasi-RCT and were not eligible for inclusion in this review.

Studies published from 2000 to up to date, in English, conducted with humans, and freely available full text online or through the University of Cambridge databases were included.

### 2.2. Search Strategy

A search of the electronic databases Scopus, MEDLINE (PubMed), Web of Science, and PsychINFO was conducted on February 2022.

The search strategy was developed utilizing key words structured from the PICOS framework and by key words used in searches by other relevant reviews. The search terms were developed initially on Scopus and adapted for use in other databases in combination with applicable database-specific filters for RCTs, humans, English language, publication date, and adults. The search term included key words describing (#1) the intervention (digital), (#2) outcome (physical activity), (#3) the study design (RCT), (#4) the condition (stroke). Gray literature was not searched. The references of all included studies were further screened to identify additional studies eligible for inclusion. Authors of included studies were contacted via email for clarifications or requests for additional information.

### 2.3. Study Screening

Records returned from the four databases were imported into the systematic review platform Rayyan [35]. With this application, reviewers can independently screen studies and utilize in-built tools to accept or reject studies and note reasons for inclusion/exclusion. Two reviewers (A.K. and S.W.) conducted screenings independently and met to discuss and resolve conflicts.

Studies eligible for full-text screening were then screened against the inclusion and exclusion criteria to ensure intervention and outcome measures aligned against the eligibility criteria. References of eligible studies were further screened for additional studies. During this stage, many studies that were technology-controlled devices or contraptions to facilitate initial stroke rehabilitation were excluded. Study protocols, feasibility and pilot studies of non-randomized design were also excluded at this point. All studies that did not meet the inclusion criteria at full-text screening were excluded and the reason for exclusion was noted in Rayyan. Eligible studies were put forward for data extraction.

### 2.4. Data Extraction

An Excel data extraction form was used to extract data from eligible studies included in the systematic review. The data extraction form had three overarching sections including study characteristics, intervention-comparator, and outcomes. The study characteristics section includes information regarding the author, country of study, number of participants included in analysis, duration of study, and intervention type/delivery. The intervention-comparator section includes descriptions and coding of the intervention and comparator such as the intervention content, delivery mode, measurements, and length and frequency of intervention. When there was more than one intervention group reported in the trial, the intervention group that received the more digitally advanced intervention was selected. Lastly, the outcomes section of the form extracted the outcome measurements, units of the measurements, and whether the outcomes for each study were conceptualized as physical activity or sedentary behaviours or both.

During data extraction, an inclusive approach was adopted to extract all reported physical activity, exercise, and sedentary behaviour outcomes. The unadjusted mean change, standard deviation, and number of participants in each arm were extracted for continuous outcomes. Both baseline and final follow-up measures were extracted for outcome data. If studies had multiple follow-up measurements, the measurement at the end of the intervention were extracted and used in the analysis. Where possible, the unadjusted data at follow-up was extracted because randomization minimizes baseline differences, thus unadjusted final values were appropriate for this review. Data reported from an intention-to-treat analysis were coded, unless there was no intention-to-treat analysis whereby available case results were extracted and selected for analysis.

### 2.5. Risk of Bias

To assess the risk of bias within individual studies, the Cochrane Risk of Bias tool Version 2 (RoB 2) was used [36]. This tool provides a framework for assessing the bias in RCTs and focuses on various aspects including trial design, conduct or implementation and reporting. For this review, the tool was used to evaluate the primary outcome of physical activity and sedentary behaviour. The risk of bias tool applied in this review was the “intention-to-treat effect” since this review aims to assess the assignment to the intervention rather than the effect of adhering to the intervention (per-protocol effect).

### 2.6. Meta-Analysis

The Review Manager (Revman) version 5.4.1 was used to run the meta-analyses [37]. Common outcome measurements were highlighted across the individual studies including the 6 min walking test (6 MWT), 10 m walk test (10 MWT), gait speed, steps per day, timed up and go test (TUG), grip strength, Wolf Motor Function Test (WMFT), Fugl-Meyer Assessment (FMA), and Barthel Index.

If an outcome had been measured both objectively and subjectively, then the objective measurement was inserted for analysis. If a study had more than one outcome measure that measured physical activity or sedentary behaviour, the measure that had a smaller standard deviation was selected as it was deemed less prone to bias and a more accurate measurement of a phenomenon effect [38].

For the summary statistic, physical activity and sedentary behaviour outcomes were both measured using different methods and scales; thus, the standardised mean difference (SMD) was selected to summarize the statistics. The standard deviations are also used to calculate the standardization of the mean differences. The SMD further expresses the size of the intervention effect in each study relative to the between-participant variability in outcome measurements observed.

The random-effects model with inverse-variance was selected as it assumes that the individual studies are estimating different, yet related, intervention effects—further accounting for heterogeneity between studies [39]. The I2 statistic was then used as a measure of heterogeneity that describes the percentage of variation across studies that is due to heterogeneity rather than chance. The Cochrane Handbook for Systematic Reviews of Intervention interprets values between 0% to 40% as low, 30% to 60% as moderate heterogeneity, 50% to 90% as substantial heterogeneity, and 75% to 100% as considerable heterogeneity [39]. If the heterogeneity is moderate or substantial, a subgroup analysis is recommended to explore the variables that account for the unexplained heterogeneity.

Data were analysed in two complementary meta-analyses—operationalized under two overarching and complementary behaviours: physical activity and sedentary behaviour. Regarding the physical activity behaviour, the direction of effect is positive since improvement is associated with higher scores or measurements such as steps per day. Thus, an effect size greater than 0 indicates the degree to which the intervention is more effective than the comparator. Concerning the sedentary behaviour, the direction of effect is negative since improvement is associated with lower scores and taking less time and/or effort to accomplish certain mobility tasks for stroke patients. Thus, an effect size less than 0 shows the degree to which the intervention is less effective than the comparator.

### 2.7. Subgroup Analysis

Four subgroup analyses were conducted based on the following categories: measurement outcome, type of digital intervention, interactivity components, and self-monitoring components. The first two subgroup analyses—outcome measurement and intervention digital delivery mode—focus on study characteristics. The next two subgroup analyses—interactivity and self-monitoring—focus on the intervention characteristics that support engagement with the mechanisms of behaviour change.

The types of digital intervention modality were categorized into four groups based on the platform, device, and mode of delivery—video game, virtual reality (VR), phone/tablet application, and monitoring device [40].

Due to the lack of explicit measures of the level of engagement with the underlying mechanisms of behaviour change, further subgroup analyses were conducted on whether or not the digital technologies consisted of functionalities that prompted two-way interactive vs. one-way non-interactive provision of information. Interactivity was coded when studies included two-way communication, rather than one-way systems that only push information to the patient [41]. Interventions were also coded as ‘interactive’ if they promoted engagement via entertainment and gamification [42].

Self-monitoring interventions were defined as interventions that recorded participants’ behaviour and provided participants with feedback on their behavioural performance, in real-time or retrospectively. This feedback further informed the provision of behavioural strategies aiming to modify or maintain their behaviour, and to therefore further support self-monitoring of the process of health behaviour change during the intervention and in a real-world setting. Self-monitoring elements were coded as operationalized by the CALO-RE taxonomy [43]. Studies not designed to support self-monitoring processes, and thus bring about behaviour change, were coded as non-self-monitoring.

## 3. Results

**Figure 1:**
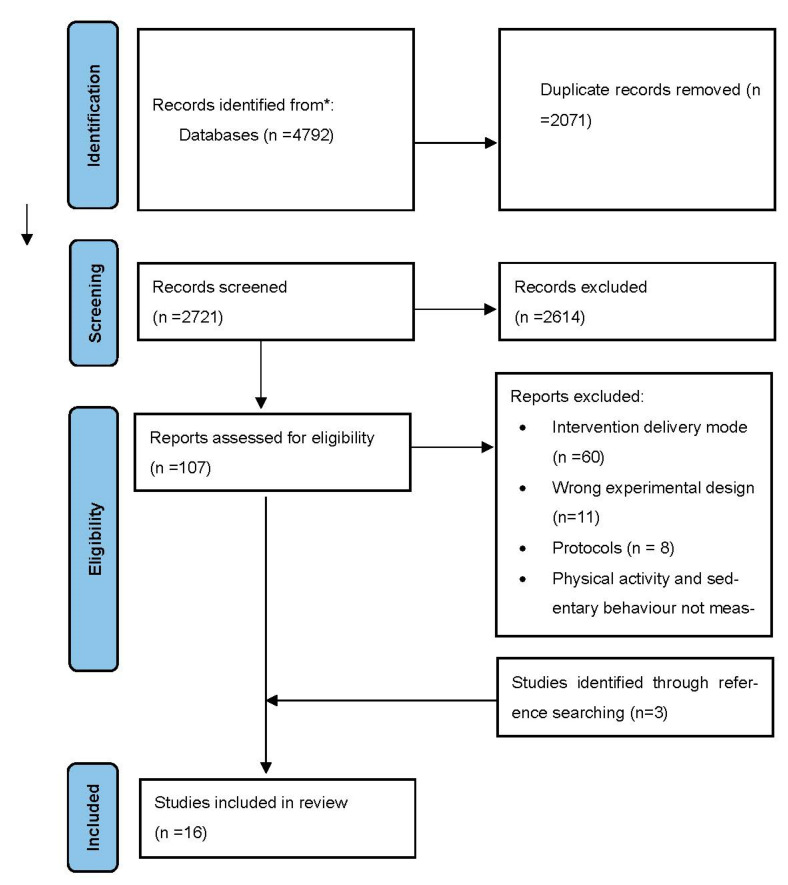
PRISMA diagram of the systematic literature review.

Overall, 4792 articles were identified from the database searches. After removing duplicates, 2721 were included for the abstract and title screening. After the abstract and title screening, 2614 studies were excluded. The remaining 107 studies were included in the full-text screening of which 13 studies met the inclusion criteria. The 94 studies were excluded with the reason for exclusion noted. Three studies were identified by reviewing the references of the 13 eligible studies. Data were extracted from a total of 16 studies eligible for quantitative analysis and narrative synthesis. The PRISMA diagram in Figure 1 details this process and the reason for exclusion at the full-text screening stage.

Studies were conducted in 11 countries—including the US, UK, Spain, Canada, Korea, Thailand, Taiwan, Republic of Slovenia, Israel, Germany, and Japan. Two studies occurred in Taiwan; two in Canada; two in Spain. There was a total of number of 799 participants, who received either the intervention or the control, included in the meta-analysis. Study duration ranged from two weeks to 12 weeks. Five studies measured and reported physical activity; six studies reported sedentary behaviour; and five studies reported measures for both physical activity and sedentary behaviours. Overall, all studies included interventions that were delivered digitally in most capacities. For the delivery mode, three used phone/tablets, five used virtual reality, four used monitoring devices, and four used video games. Heron et al. and Chung et al. had multiple intervention arms, the intervention most digitally advanced was selected [44,45]. Chung et al., Givon et al., and Saposinik et al. had multiple follow-up measurements; the time point that was at the end of the intervention was at the end of the intervention was selected as the corresponding follow-up value and measure [46,47,48].

### 3.1. Risk of Bias Assessment

**Figure 2:**
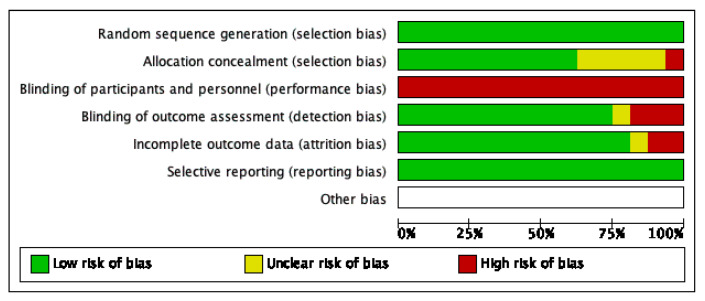
Summary of risk of bias assessment for each domain as a percentage of included studies.

The Cochrane Risk of Bias tool for the 16 included studies is shown in Figure 2. The risk of bias varied due to intervention behavioural components, study design, and outcome measurements. All studies had a low-risk of bias in the randomization process since they all reported validated randomization methods. All studies had a high risk of performance bias since it was not possible for this kind of interventions to blind participants to the group they were randomized to. The selection bias from allocation concealment was low for 10 studies, unclear for five, and high for one study. Detection bias measured by the blinding of assessors was low for 12 studies, unclear for three studies, and high for one study. For attrition bias, there were nine studies that had low risk, one study with unclear risk, and six studies with high risk. High-risk studies had outcome data missing for more than 10% of randomised participants, sensitivity or imputation analyses were not performed, or no evidence that the result was not biased by the missing outcome data was provided. All studies had low selection risk of bias since it was explicitly reported that it was intended to measure certain outcomes and reported those related outcomes.

### 3.2. Physical Activity Behaviour

**Figure 3:**
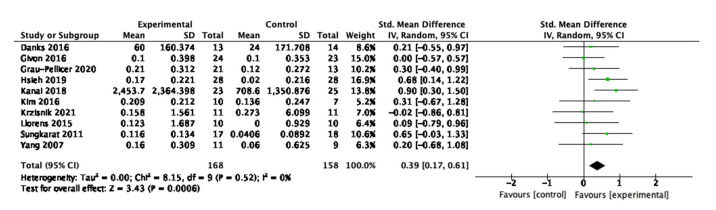
Meta-analysis results for physical activity.

The meta-analysis for physical activity is displayed in Figure 3. Ten out of the 16 studies reported physical activity outcome measurements and were included in the analysis. The intervention had a moderate and statistically significant effect on improving physical activity behaviour (SMD = 0.39, 95% CI 0.17, 0.61, N = 326, *p* < 0.001). Heterogeneity among the studies was low and not statistically significant (Tau2 = 0.00, Chi2 = 8.15, df = 9, *p* = 0.52, I2 = 0%, test for overall effect Z= 3.43, *p* < 0.001), suggesting that the effect of the intervention did not vary considerably among the individual studies.

### 3.3. Sedentary Behaviour

**Figure 4:**
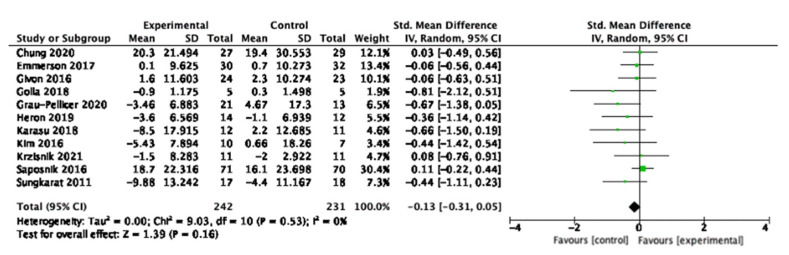
Meta-analysis results for sedentary behaviour.

The meta-analysis for sedentary behaviour is displayed in Figure 4. Eleven of the 16 studies reported sedentary behaviour outcome measurements and were included in the analysis. The data shows that although there was a trend towards a small and potentially clinically significant decrease in sedentary behaviour, the intervention did not have a statistically significant effect on changing overall sedentary behaviour (SMD = −0.13, 95% −0.31, 0.05, N = 473, *p* = 0.53). The heterogeneity among the studies was low and not statistically significant (Tau2 = 0.00, Chi^2^ = 9.03, df = 10, *p* = 0.53, I_2_ = 0%, test for overall effect Z = 1.39, *p* = 0.16).

### 3.4. Publication Bias

**Figure 5:**
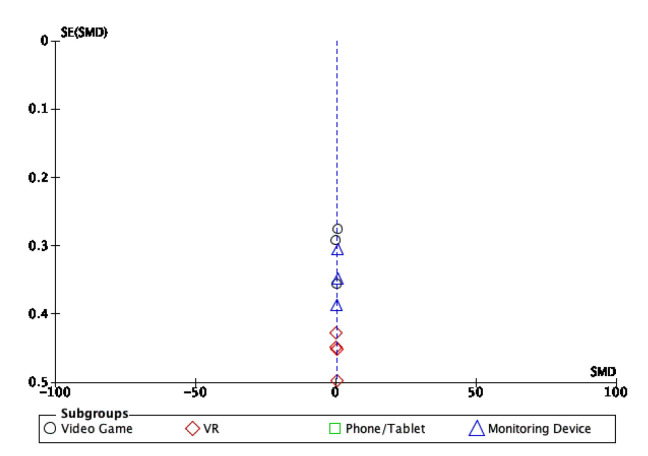
Funnel plot of the standardised mean difference against the standard error for physical activity.

**Figure 6:**
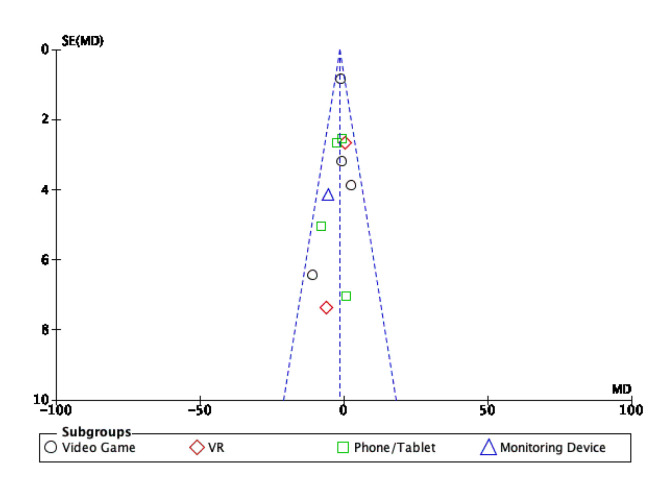
Funnel plot of the standardised mean difference against the standard error for sedentary behaviour.

Assessment for publication bias and small study effect was conducted using a funnel plot. The funnel plot for physical activity is displayed in Figure 5 and for sedentary behaviour in Figure 6. The funnel plot displayed for physical activity and sedentary behaviour appears to be symmetrical.

Several studies were excluded from the meta-analysis because the outcome measures were not consistent and comparable to other included studies. For example, two studies reported the median rather than the mean [49,50]; however, their results align with what is shown from this review’s meta-analyses suggesting the digital interventions may be effective.

### 3.5. Subgroup Analyses

Subgroup analyses were conducted to disentangle complexity and understand whether certain elements and components of the digital intervention could potentially explain best the observed intervention effectiveness. Although the studies had low heterogeneity, and usually subgroup analyses are not recommended, we performed these analyses to describe generic trends, and rather definite effects, and to better inform the evidence-base and recommendations about the characteristics of the most effective interventions. The subgroup analyses were performed for exploratory reasons to inform our narrative analysis regarding the directions for future intervention development.

### 3.6. Outcome Measurements

**Figure 7:**
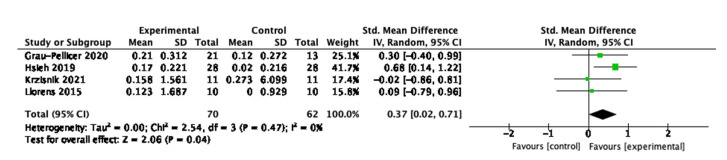
Subgroup analysis for the 10 m walk test.

Regarding the physical activity behaviour, three studies measured number of steps per day [47,51,52]; four studies measured walking distance using the 10 m Walk Test [53,54,55,56]; four studies measured walking time using the 6 min walking test [45,52,53,54]; and four studies measured gait speed [45,47,57,58]. There was a trend suggesting the 10 m walk test capturing the most rigorous and positive intervention effects on improving behaviour, which is displayed in Figure 7. The standardised mean difference is 0.37 (95% CI: 0.02, 0.71) with a *p*-value of 0.04. This finding suggests that digital interventions are be more effective at supporting improvements in walking behaviour, and specifically efficiency of steady-state walking speed, manifesting significant improvements in stroke patients’ physical health.

**Figure 8:**
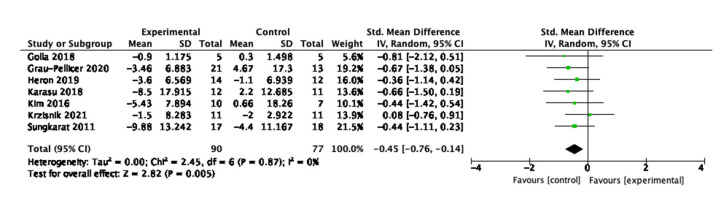
Subgroup analysis for the timed up and go test.

Concerning the sedentary behaviour; seven studies measured time-duration of interrupted sitting behavior using the timed up and go test [44,45,53,54,58,59,60]; two studies measured mobility strength using the grip strength test [47,61]; two studies measured mobility function using the Wolf Motor Function test [48,61]; and three studies measured mobility independence using the Barthel Index [46,48,54]. The analysis suggested interventions having a significant effect at reducing sedentary behaviour as measured by the timed up-and-go test, which is displayed in Figure 8. The standardised mean difference is −0.45 (95% CI: −0.76, −0.14) with a *p*-value of 0.005. This evidence suggests that digital interventions are effective at reducing sedentary behaviour, indicating significant impact of the interventions at preventing risks for falls after stroke.

### 3.7. Type of Digital Intervention

**Figure 9:**
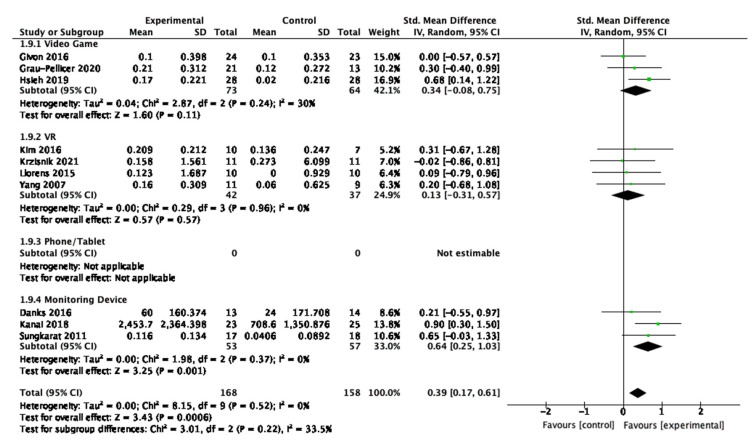
Subgroup analysis for the type of digital interventions that measured physical activity outcomes.

For physical activity, there were three studies that used video game interventions [47,54,55]; four that used immersive virtual reality [45,53,56,57]; none that were phone/tablet devices; and three studies had monitoring devices [51,52,58]. There was a trend for the monitoring device interventions showing a moderate to large overall positive effect (SMD = 0.64, 95% CI 0.25, 1.03, *p* = 0.001); whereas none of the other digital interventions had a significant effect as shown in Figure 9.

**Figure 10:**
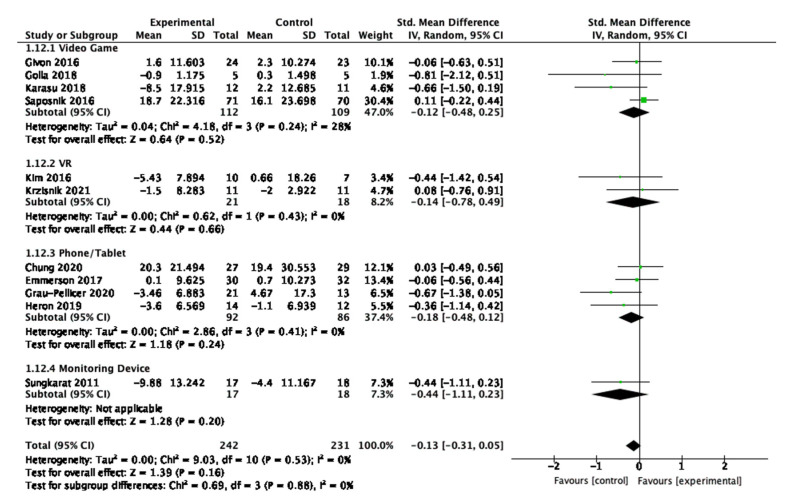
Subgroup analysis for the type of digital interventions that measured sedentary behaviour outcomes.

For sedentary behaviour, there were four studies that used video games [47,48,59,60]; two that used immersive virtual reality [45,53]; four that used phone/tablet devices [44,46,54,61]; and one that used a monitoring device [58]. There were no statistically significant tendencies observed that could describe what the most effective interventions are to improve sedentary behaviour, based on the digital technology utilised to facilitate the intervention. This is displayed in Figure 10.

### 3.8. Digital Interactivity

**Figure 11:**
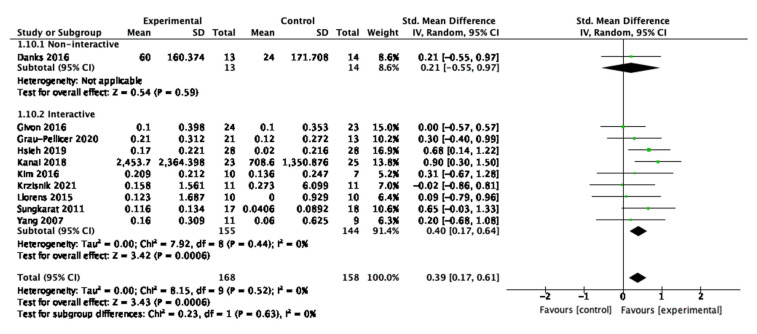
Subgroup analysis for participant interactivity with the digital interventions that measured physical behaviour outcomes.

For physical activity, one study was categorized as non-interactive, and nine studies were categorized as interactive. Overall, effective interventions included interactive elements to engage patients with their treatment and improve physical activity, as shown in Figure 11. For the interactive interventions, the SMD is 0.04 (95% CI 0.17, 0.64) with a *p*-value of 0.0006. Hsieh et al. is the study with the greatest weight at 16.9%.

**Figure 12:**
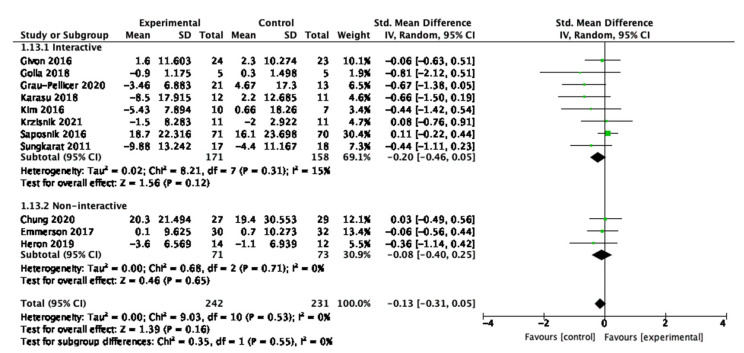
Subgroup analysis for participant interactivity with the digital interventions that measured sedentary behaviour outcomes.

For sedentary behaviour, eight studies were interactive, and three studies were non-interactive. Neither the interactive nor the non-interactive studies were statistically significant impactful in the overall effect as shown in Figure 12.

### 3.9. Self-Monitoring

**Figure 13:**
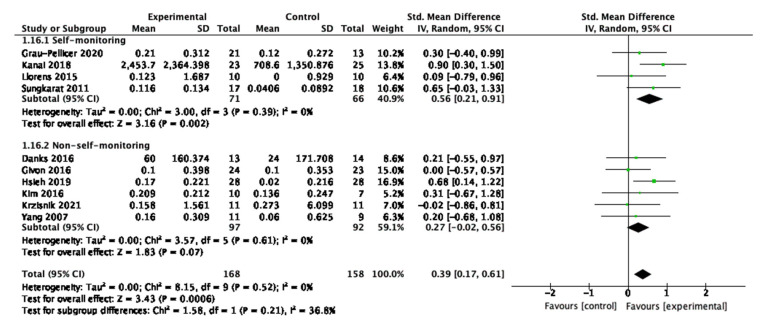
Subgroup analysis for self-monitoring of digital interventions that measured physical activity outcomes.

For physical activity, four studies consisted of self-monitoring intervention strategies and six studies had no strategies to support self-monitoring. Overall, there was a tendency for self-monitoring interventions to have moderate and large impact on improving behaviour as shown in Figure 13. For the self-monitoring interventions, the SMD is 0.56 (95% CI 0.21, 0.91) with a *p*-value of 0.002.

**Figure 14:**
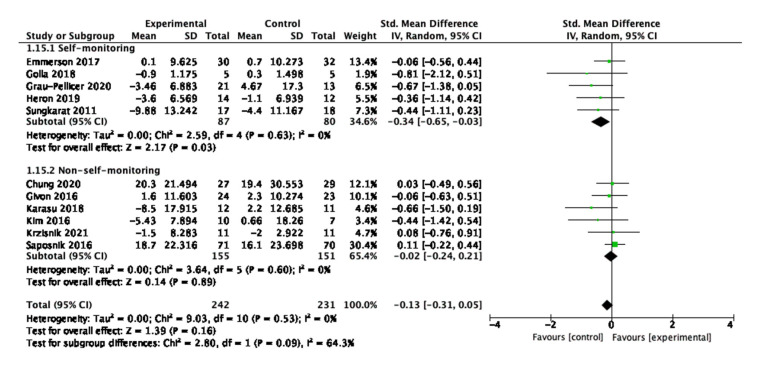
Subgroup analysis for self-monitoring of digital interventions that measured sedentary behaviour outcomes.

For sedentary behaviour, five studies had self-monitoring interventions and six studies had non-self-monitoring interventions. The self-monitoring interventions significantly explained the overall effectiveness compared to those not including such components, as shown in Figure 14. For the self-monitoring interventions, the SMD is −0.34 (95% CI −0.65, −0.03) with a *p*-value of 0.03. Emmerson et al. is the study with the greatest weight at 13.4%.

Overall, for changes in both physical activity and sedentary behaviours, interventions underpinned by self-monitoring elements were more likely to explain effectiveness. This finding suggests that digital interventions consisting of self-monitoring components tend to be more effective in promoting physical activity and reducing sedentary behaviour when they consist of self-monitoring components. The study with the largest weight for physical activity measures is Kanai et al., which uses monitoring devices, and for sedentary behaviour outcome measures is Emmerson et al. which uses a phone/tablet device application. Both these studies, and those contributing to the characteristics of the most effective interventions, were conducted in adjunct to usual care rehabilitation programs, and the digital self-monitoring component was followed up by in-person communication advice with a health care professional. These types of interventions may have greater potential for self-monitoring functionalities that provide ongoing feedback for modifying or maintaining behaviour change, and might also be implementable in adjunct to usual care post-stroke treatment programmes.

### 3.10. Sensitivity Analysis

Post hoc sensitivity analyses were conducted to investigate the effect of the sample size of individual studies on the physical activity and sedentary behaviour outcomes. This was to improve current knowledge and understanding about whether smaller sample sized studies were adequately powered to detect an effect or whether small sample sizes were biasing the results [62]. The median sample size was calculated for the studies, and it determined the threshold for small and large sample sizes for this review. Of the ten studies included for sensitivity analysis of physical activity, the median sample size was 30. Studies with small sample sizes had 30 or fewer participants; studies with large sample sizes had greater than 30. For sedentary behaviour, the median, and therefore threshold, was a sample size of 34.5.

**Figure 15:**
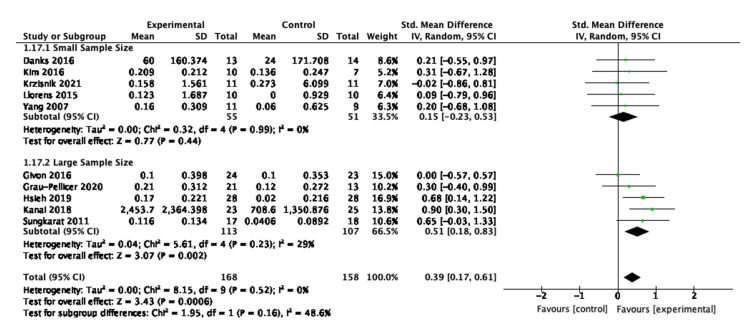
Sensitivity analysis for physical activity.

For physical activity, five studies had small sample sizes and five had large sample sizes as shown in Figure 15. The effect size was larger in the large studies (SMD = 0.51, 95% CI 0.18, 0.83, *p* = 0.002), suggesting that the interventions are feasible and the observed effect scalable. The results show that there was no significant variability of the effect within the small sample sized studies (I_2_ = 0%, *p* = 0.99) and moderate non-significant heterogeneity within the large sample sized studies (I_2_ = 29%, *p* = 0.002).

**Figure 16:**
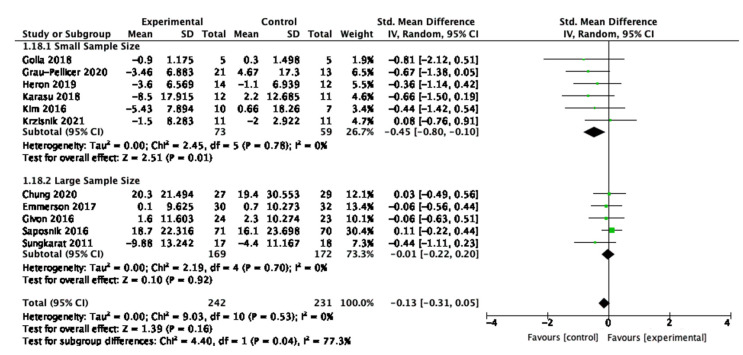
Sensitivity analysis for sedentary behaviour.

For sedentary behaviour, five studies had a small sample size, and six studies had a large sample size as shown in Figure 16. The effect size was larger in the smaller studies (SMD = −0.45, 95% CI −0.80, −0.10, *p* = 0.01), suggesting intervention acceptability and potential feasibility to detect effectiveness. There was low and non-significant variability of the effect within the individual subsets. However, the test for difference between the subsets was significant (I2= 77.3%, *p* = 0.04)

## 4. Discussion

### 4.1. Principal Findings

This systematic literature review identified 4792 studies, of which 2721 underwent abstract and title screening, 107 studies full-text screening and 16 randomised controlled trials with 799 participants were included in the meta-analysis. The quantitative analysis proved that digital interventions underpinned by self-regulation principles are effective at changing increasing walking behaviour (SMD = 0.39, 95% CI 0.17, 0.61, n = 326, *p* < 0.001) and reducing sedentary time (SMD = −0.45, 95% CI: −0.76, −0.14) in stroke survivors, proved by objective measures of the behaviour.

This review is the first rigorous evidence base to quantify the effectiveness of digital interventions to improve physical activity and reduce sedentary behaviour, for stroke patients. These important results align with previous studies in the literature that also find digital interventions potentially effective in supporting physical activity but not specifically in patients after stroke [26,27,40,63]. Moreover, there has been a lack of evidence that specifically investigates the impact of digital interventions on sedentary behaviour in stroke populations. One review, published in 2021, did not find any studies reporting measures of sedentary behaviour [27]. Another review focuses on the behaviour change techniques of interventions that target sedentary behaviour in general and heterogeneous clinical populations rather than the effectiveness in patients after stroke [64]. However, the growing attention towards reducing sedentary behaviour is important to develop interventions that explicitly address inactivity in this patient group. Especially due to the older demographic of stroke survivors and the debilitating effects of stroke, interventions that target sedentary behaviour can be more inclusive of functionality and mobility as a stepping stone to physical activity and exercise behaviours to better support patients with stroke treatment. Moreover, synergistically these behaviours also may yield benefits for the prevention of further health complications and reduction of health care costs.

Although heterogeneity was low, four subgroup analyses—outcome measure, type of digital intervention, interactivity, and self-monitoring—suggest avenues and provide directions for the most effective intervention characteristics, and form suggestions for future intervention development and clinical practice. The additional critical evaluation and analyses, helped to disentangle complexity and better understand the elements and components of interventions that contributed to its effectiveness. The findings suggest interventions to produce statistically significant improvements in physical activity behaviour, when utilising the 10 m walk test that is highly used in usual care clinical practice for stroke rehabilitation. For sedentary behaviour, the critical intervention evaluation suggests most effective interventions to utilise the ‘timed up and go test,’ which is a clinical performance-based measure of lower extremity function, mobility, and risk of falls, and is a highly recommended test in clinical practice for stroke treatment and rehabilitation programmes. This finding suggests that this evidence is implementable in usual care practice. Additionally, self-monitoring interventions that encouraged interactivity, with or without the use of monitoring feedback devices, had a significant overall effect on changing health behaviour. This evidence provides directions about the effective and replicable methods to support stroke treatment, across levels of the condition and populations. Sensitivity analysis found that study size may explain some of these trends weighted by the power of the available evidence to detect differences in the effect sizes.

### 4.2. Strengths and Limitations of this Review

The first strength of this review is that it fills an important gap in the existing literature and contributes to an important field of public health research, especially with the increasing popularity of digital medicine due to the COVID-19 pandemic and rapidly evolving technological advancements in health care. The rationale for this systematic review was rooted in the public health significance of stroke and the importance of rigorous digital health interventions to access and improve physical activity and reduce sedentary behaviour in this population. This research question had not been investigated previously.

One of the first steps in conducting this review was composing a protocol for the systematic review and meta-analysis. The protocol outlined the research question using the PRISMA guidelines and PICOS framework as detailed in the methodology. Composing and registering a protocol on PROSPERO ensured that there were no deviations from the aims and objective and reduced bias for the stages of the review process, methods, and the outcomes reported.

A systematic search of the literature was conducted in four electronic databases using a pre-specified detailed search strategy that encompassed search terms used in similarly related reviews. The references of included studies were further searched to identify eligible studies that may have been missed from the databases. The screening process was conducted thoroughly and independently by the two reviewers with the Rayyan software.

Moreover, most of the outcome measurements selected for the meta-analysis were objective measures of the behaviour utilised in usual care clinical practice; thus, the evidence of this review is robust and implementable. The subjective categorizations for subgroup analyses were conceptualised using existing frameworks of interactivity and self-monitoring components of digital interventions. These categorizations were used to best systematically code intervention components. Lastly, the strong lack of heterogeneity of the included studies displays the rigor of the evidence.

One of the limitations of this review is that the gray literature was not searched which can leave the review prone to publication bias. However, the in-depth literature search and the thorough assessment of the risk of bias and the quality of the controlled trials, as well as the stastistical exploration of potential bias, provides us with confidence that all rigorous evidence has been identified and synthesized critically to inform our research aims.

Another limitation is that some studies that did not provide the outcome data in comparable format and thus were excluded. This could lead to bias in the results; however, the results and suggested directions from the excluded studies did not differ from the overall effect found in our review, thus the risk of publication bias has been mitigated. Furthermore, we have investigated four out of the many study characteristics and intervention components of an intervention guided by theoretical and methodological frameworks to disentangle complexity and provide the directions for future interventions, practice, and research.

### 4.3. Strengths and Limitations of Included Studies

A major strength is the RCT study design of the eligible studies. The randomization of participants to the intervention and comparator arm minimizes potential selection bias and confounding factors that are common in observational studies. An RCT is the only study design that can account for both known and unknown confounders. Thus, with randomization, there is an equal distribution of potential confounding factors in both intervention and control groups and the effect size should be largely unbiased.

The included studies were further delivered in real world clinical settings rather than in a laboratory setting. Thus, the evidence synthesized from the studies can inform clinical practice and research about the use of digital interventions to improve physical activity and reduce sedentary behaviour, and sustain behaviour change in adjunct to usual care and rehabilitation programs.

An important limitation of the individual studies is the variation in duration and intervention components among the studies. Studies used various behavioural components and were in varying settings (at-home in adjunct to or in-clinic, tailored to the stage of stroke). However, heterogeneity was measured and suggested that these elements might not be statistically significant in the available evidence-base.

Studies also had a high risk of bias for performance bias since participants could not be blinded and a handful had a large risk of detection and attrition bias. However, the most rigorous trials in this review provided evidence to support that digital interventions are effective solutions for stroke patients in promoting physical activity and reducing sedentary behaviour. This review is the first to analyse all available evidence and provide the most comprehensive and robust evidence base to inform future clinical work, research, and policy. A simple analysis of funnel plots further provides a useful test for the likely presence of bias in meta-analyses [65].

## 5. Conclusions

In this review, we systematically searched and identified all available evidence in RCTs on digital health interventions supporting physical activity and reducing sedentary behaviour in adults who have experienced a stroke. The results of the meta-analyses suggest that digital interventions are effective at promoting physical activity; however larger and scalable studies are necessary to impact and more effectively change behaviour in patients after stroke. Based on the findings of this review, there is a great potential for digital interventions to be applied in practice; however, future research in this area should be to increase sample sizes, include more objective measures of intervention engagement, and break down the complexity of current interventions to confidently attribute the intervention’s effectiveness to certain behavioural components and corresponding mechanisms of action. Eventually with results from future larger, more robust studies, health policies can further bridge the research into real-world and impact.

## Data Availability

Not Applicable.

## Appendix A

**Table A1:** PICOS framework.

PICOS Categories	Description
Population	Adults (≥18 years old) with a history, explicit diagnosis of stroke
Intervention	Two-way or one-way interactive digital behavioural interventions introduced or facilitated by healthcare providers in adjunct to rehabilitation programs, that aim to promote physical activity and disrupt sedentary behaviour—including automated Short Message Service (SMS), email, web-based applications, telephone calls, video, virtual-reality, tablet or phone applications (apps), digital games, and integrated monitoring tools such as wearable sensors or accelerometers Technology-controlled interventions such as robot-assisted devices that assist to control bodily movement at early stages of stroke and stroke rehabilitation will be excluded
Comparator	Usual care, minimal intervention such as in-person and face-to-face sessions, or a digital intervention not supporting physical activity/sedentary behaviour change
Outcomes	Changes in physical activity and/or sedentary behaviour which include, but are not limited to, minutes of moderate-to-vigorous physical activity, number of steps, distance travelled, minutes of walking, independent functionality, mobility, and are measured by self-reports or objectively, for example by a pedometer, mobile phone sensors, accelerometer, or other wearable sensors
Design of Studies	Randomised controlled trials
